# Gonioscopy-Assisted Transluminal Trabeculotomy following Failed Glaucoma Surgery in Primary Congenital Glaucoma: One-Year Results

**DOI:** 10.1155/2023/6761408

**Published:** 2023-06-01

**Authors:** Abdelrahman M. Elhusseiny, Reem M. Aboulhassan, Yasmine M. El Sayed, Ghada I. Gawdat, Hala M. Elhilali

**Affiliations:** ^1^Department of Ophthalmology, Kasr Al-Ainy Hospitals, Cairo University, Cairo, Egypt; ^2^Department of Ophthalmology, Harvey and Bernice Jones Eye Institute, The University of Arkansas for Medical Sciences, Little Rock, AR, USA

## Abstract

**Purpose:**

To evaluate the one-year outcomes of gonioscopy-assisted transluminal trabeculotomy (GATT) in primary congenital glaucoma (PCG) with a history of prior glaucoma surgery.

**Methods:**

A retrospective chart review was performed to identify all PCG patients ≤ 16 years who underwent GATT surgery at Cairo University Children's Hospital from January 2016 to March 2022. Pre- and postoperative intraocular pressure (IOP) and glaucoma medications were collected at 1, 3, 6, 9, 12, and last follow-up visits. Success was defined as IOP ≤ 21 mmHg without (complete) or with (qualified) glaucoma medications at the last follow-up.

**Results:**

Seven eyes of 6 subjects were included in the study. The mean IOP was statistically significantly reduced from 25.7 ± 5.9 mmHg preoperatively to a mean IOP of 12 ± 1.5 mmHg (*P* = 0.001) at 12 months and 11.5 ± 1.2 mmHg (*P* = 0.001) at the last follow-up visit. Six eyes (85.7%) achieved complete success, and one eye (14.2%) achieved qualified success. No patients required further glaucoma procedures. No serious intra- or postoperative complications were identified.

**Conclusions:**

Our early experience highlights that GATT can be performed as an alternative procedure before considering conjunctival or scleral glaucoma surgeries.

## 1. Introduction

Pediatric glaucoma is an important cause of irreversible vision loss worldwide [1, 2]. Management of primary congenital glaucoma (PCG) is mainly surgical. Angle-based surgeries are considered the standard of treatment, whereas more invasive surgeries, such as trabeculectomy and tube shunts, are usually resorted to when angle surgery fails [3, 4]. Gonioscopy-assisted transluminal trabeculotomy (GATT) was recently introduced by Grover et al. [5]. It allows the cannulation of Schlemm's canal (SC) using a suture or microcatheter through a corneal incision [5, 6]. There has been growing evidence in the literature supporting its efficacy in adult glaucoma; however, few studies have evaluated its efficacy in PCG [6–10]. The purpose of this study is to evaluate the efficacy and outcomes of GATT in a PCG cohort who underwent previous glaucoma surgeries.

## 2. Methods

A retrospective chart review was performed to identify all PCG patients ≤ 16 years who underwent GATT surgery at Cairo University Children's Hospital from January 2016 to March 2022. Excluded patients were those with childhood glaucoma other than PCG, PCG patients who had not undergone a glaucoma procedure before GATT, and those with < 12 months of follow-up. Data collected included age at the time of GATT surgery, previous ocular surgeries, gender, laterality, and family history including parental consanguinity, pre- and postoperative vertical cup-to-disc ratio (C/D), and surgical details including the extent of GATT, intra- or postoperative complications, and need for additional glaucoma procedures. In addition, pre-and postoperative intraocular pressure(IOP) and glaucoma medications at 1, 3, 6, 9, 12, and last follow-up visits were collected. Visits within 2-3 days of the first month of the surgical date were tabulated as the whole number of follow-up month (e.g., 1 month+/-3 days were tabulated as a 1-month postoperative visit) and after that and within 2 weeks for the 3-,6-, and 12-month visits (e.g., 3 months +/-2 weeks were tabulated as a 3-month postoperative visit).

The IOP was measured in all patients using Perkins® handheld applanation tonometry during office visits after applying topical anesthesia. For uncooperative children, IOP measurements were obtained after administrating chloral hydrate. Success was defined as IOP ≤ 21 mmHg without (complete) or with (qualified) glaucoma medications at the last follow-up and without the need of additional glaucoma procedures or development of vision-threatening complications.

The study was approved by the ethics committee of Cairo University (approval number: MD-189-2021) and adhered to the tenets of the declaration of Helsinki. Informed consent for the surgical procedure and inclusion in the study was obtained from the patients' legal guardians.

### 2.1. Statistical Analysis

Data were entered using Microsoft Excel 2016 and analyzed using the Statistical Package For Social Sciences (SPSS Inc., Chicago, IL, USA, version 24). Quantitative data were expressed as median, mean ± standard deviation, and range. A *p* value <0.05 was considered statistically significant.

### 2.2. Surgical Technique

**Figure 1:**
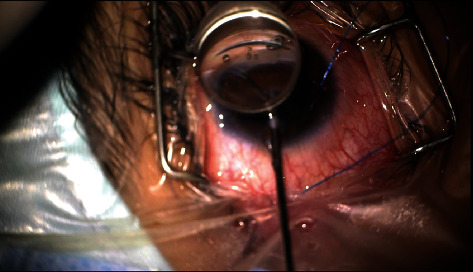
The figure shows pulling the distal tip of a 5/0 polypropylene suture to exert traction on the suture creating a trabeculotomy in a primary congenital glaucoma patient with a previous Ahmed glaucoma valve implantation.

All surgeries in this series were performed using a 5/0 polypropylene suture. The suture end was first blunted with cautery. A temporal corneal incision was created. Under direct gonioscopic view, a nasal goniotomy was performed using a 23 gauge (G) microsurgical blade, and the suture was grasped with 23G forceps and introduced into SC through one end of the goniotomy incision. The suture was then advanced through SC until the distal tip of the suture was retrieved through the opposite end of the goniotomy incision. Both suture ends were then held and pulled simultaneously, creating circumferential trabeculotomy (Figure 1) (video (available here)). All surgeries were performed by one surgeon (Y.M.E). Postoperatively, all patients have received a combination of topical antibiotic/steroid with gradual tapering over 2 weeks. The GATT procedure was performed only in eyes with clear corneas to enable visualization and without extensive peripheral anterior synechiae on gonioscopy.

## 3. Results

Seven PCG eyes (6 patients) underwent GATT surgery with at least 12 months of follow-up. All patients had undergone previous glaucoma surgery (Table 1). Four patients were males. Parental consanguinity was identified in 2 patients. None of the patients had a positive family history of childhood glaucoma. One patient (eyes #5 and #6) had bilateral GATT one week apart. In 5 eyes, a circumferential 360° GATT was performed. All eyes had a clear cornea and an immature angle with patchy peripheral anterior synechiae from the previous procedures. The extent of peripheral anterior synechiae was not documented in the patients' records, but eyes with extensive peripheral anterior synechiae did not undergo GATT. In one eye with a previous goniotomy, around 70 degrees of the nasal angle showed missing areas of the pigmented trabeculum with patchy peripheral anterior synechiae. Another eye with unknown previous surgery showed scarring over 2 clock hours of the nasal angle, suggesting a previously attempted goniotomy.

The mean age at the time of GATT surgery was 62.8 ± 29.7 months (median; 60 months). The median follow-up was 13.5 months (mean: 15.7 ± 4 months, range; 12-24.1 months). The mean IOP was statistically significantly reduced from 25.7 ± 5.9 mmHg preoperatively to a mean IOP of 15 ± 2.2 mmHg (median; 14 mmHg) at 1 month (*P* = 0.01), 13 ± 1.6 mmHg (median; 12 mmHg) at 3 months (*P* = 0.005), 15.2 ± 3.2 mmHg (median; 16 mmHg) at 6 months (*P* = 0.02), 12.1 ± 1.8 mmHg (median; 12 mmHg) (*P* = 0.001) at 9 months, 12 ± 1.5 mmHg (median; 12 mmHg) (*P* = 0.001) at 12 months, and 11.5 ± 1.2 mmHg (median; 12 mmHg) (*P* = 0.001) at the last follow-up visit.

The mean number of glaucoma medications was statistically significantly reduced from 1.5 ± 1.1 (median; 2) preoperatively to a mean of 0 ± 0 at 1 month (*P* = 0.01), 0 ± 0 at 3 months (*P* = 0.01), 0 ± 0 (median; 0) at 6 months (*P* = 0.01), 0.14 ± 0.34 (median; 0) (*P* = 0.03) at 9 months, 0.14 ± 0.34 mmHg (median; 0) (*P* = 0.03) at 12 months, and 0.14 ± 0.34 mmHg (median; 0) (*P* = 0.03) at the last follow-up visit.

Six eyes (85.7%) achieved complete success, and 1 eye (14.2%) achieved qualified success. No patients required further glaucoma procedures. No serious intra- or postoperative complications were identified. Mild hyphema developed in 3 eyes (42.8%), which resolved in the first seven days postoperatively on conservative treatment.

## 4. Discussion

In the current study, we evaluated the outcomes of GATT in PCG patients who had undergone previous glaucoma surgeries with a mean follow-up of 15.7 months. We found that GATT effectively reduced IOP and the number of glaucoma medications at all follow-up time points. Limited literature has been published about the outcomes of GATT in PCG patients [6–9]. A study by Chen et al. [7] evaluated risk factors for failure following GATT in a young cohort (88 patients) with a mean age of 30.5 ± 29 years at the time of surgery. Although their study included 17 PCG patients, the authors did not specifically analyze this subgroup of patients and did not report any information regarding the history of previous glaucoma surgeries, degree of IOP reduction, or reduction in glaucoma medications, and the median follow-up was short (7.4 months). We reviewed the literature and found that the efficacy of GATT in PCG was evaluated only in 8 patients. It was first reported by Grover et al. [6], who evaluated GATT in 4 PCG eyes retrospectively. They found that the mean IOP decreased from 31 ± 10.4 to 19.5 ± 4.3 mmHg, and the mean number of medications decreased from 1.5 ± 1.6 to 0. They did not report whether these patients had a history of glaucoma surgeries before GATT. A case series by Lehmann-Clarke et al. [8] reported the outcome of microcatheter-assisted GATT in four eyes with PCG, two of which had undergone previous goniotomy. The mean IOP was reduced from 24.2 ± 4.4 mmHg on 2.5 ± 0.8 medications preoperatively to 14.8 ± 2.2 mmHg on 0 glaucoma medications postoperatively. A case report by Song et al. [9] reported the success of GATT in 2 PCG eyes with no history of previous glaucoma surgery. They reported consistent IOP reduction postoperatively over 6 months of follow-up. These reports suggest that GATT is effective in reducing IOP in PCG patients, but they did not evaluate the group of patients with a prior history of glaucoma surgery except for 2 eyes in the cohort by Lehmann-Clarke et al. [8].

In our series, we found that GATT was effective in PCG patients with a history of previous glaucoma surgeries. This is similar to the findings by Shi et al. [11] and Hu et al. [12], who reported successful IOP reduction after circumferential trabeculotomy in PCG patients with failed glaucoma surgeries with success rates of 77% and 80%, respectively. However, their surgical techniques (ab-externo microcatheter-assisted trabeculotomy) were different from our study (ab-interno suture GATT).

In eyes with previous trabeculectomy, the suture passage was not interrupted at the site of the previous sclerostomy, and 360-degree cannulation of SC was possible in all cases. This is most likely because trabeculectomy in children is performed anteriorly compared to adult eyes to avoid ciliary body injury in eyes with stretched limbal anatomy [13]. In eyes that previously underwent trabeculotomy, although SC scarring was expected to interrupt the suture passage, the suture was threaded successfully around the angle. This was also noted in a study by Shi et al. [11] in which a 360-degree microcatheter-assisted trabeculotomy was performed in eyes that previously underwent angle surgery. One explanation could be that the angle was missed in the first surgery, with ab-externo trabeculotomy being a relatively blind procedure. In one eye with a previous goniotomy, the scarred nasal part of the angle was incised using the MVR blade until an intact part of the canal was reached (around 70-90 degrees), and the suture end was then inserted.

Although circumferential angle surgery has been shown in many studies to be associated with a higher success rate than traditional 120–180-degree goniotomy or trabeculotomy, still, the latter procedures have a fair success rate ranging from 31 to 70% in studies that compared them to circumferential trabeculotomy [14]. In the two eyes that did not complete a circumferential angle incision, at least 270 degrees of the angle were successfully incised, which is still more than what is achieved with conventional trabeculotomy/goniotomy.

The current study is limited by its retrospective nature and small sample size but highlights the advantages of this novel technique. However, a significant limitation is the lack of a control group for comparison (e.g., conventional angle surgery such as goniotomy or standard metal probe trabeculotomy or microcatheter-assisted trabeculotomy). Therefore, future prospective comparative studies with larger sample sizes and longer follow-ups are needed.

In conclusion, we present a case series evaluating GATT after failed glaucoma surgeries in PCG. GATT was effective in our cohort over a 12-month follow-up. Our early experience highlights that GATT can be performed as an alternative procedure before considering conjunctival or scleral glaucoma surgeries.

## Figures and Tables

**Table 1:** Baseline and postoperative clinical information of the whole cohort.

Eyes	Gender	Age at time of GATT	Preoperative cup-to-disc ratio	Previous glaucoma surgeries	Laterality	Preoperative IOP (mmHg)	Preoperative glaucoma medications	Preoperative VA (in decimals)	Extent of GATT	IOP at final follow-up (mmHg)	Glaucoma medications at final follow-up	Duration of follow-up (months)	Final VA (in decimals)
#1	M	10 years	0.9	Unknown glaucoma surgery at age of 6 months, 2 Ahmed GDD, and CPC	Left	24	1	HMGP	270	10	1	13.6	HMGP
#2	M	5 years	0.8	Ab-externo 180 trabeculotomy	Right	36	0	F&F	360	12	0	12	0.4
#3	F	3 years	0.5	Unknown glaucoma surgery at the age of 10 months at OH (? goniotomy)	Right	28	0	F&F	300	11	0	14.9	F&F
#4	F	1 year and 5 months	0.7	Trabeculectomy at OH	Right	28	2	F&F	360	12	0	24.1	F&F
#5	M	6 years	0.3	Trabeculectomy at OH	Right	16	3	0.2	360	10	0	13.4	0.5
#6	M	6 years	0.3	Trabeculectomy at OH	Left	20	3	0.3	360	14	0	13.4	0.7
#7	M	5 years	0.8	Goniotomy	Left	28	2	F&F	360	12	0	19.1	0.15

GATT: gonioscopy-assisted transluminal gonioscopy; IOP: intraocular pressure; GDD: glaucoma drainage device; CPC: cyclophotocoagulation; OH: outside hospital; M: male; F: female; VA: visual acuity; HMGP: hand motion good projection; F&F: fix and follow.

## Data Availability

The data can be shared with a reasonable request to the corresponding author due to patient confidentiality.

## Supplementary Materials

Supplementary Materials Video: surgical video showing steps of gonioscopy-assisted transluminal trabeculotomy in a primary congenital glaucoma patient.

## References

[1] Gilbert C., Foster A. (2001). Childhood blindness in the context of VISION 2020--the right to sight. *Bulletin of the World Health Organization*.

[2] Tam E. K., Elhusseiny A. M., Shah A. S., Mantagos I. S., VanderVeen D. K. (2022). Etiology and outcomes of childhood glaucoma at a tertiary referral center. *Journal of AAPOS*.

[3] Chen A., Yu F., Law S. K., Giaconi J. A., Coleman A. L., Caprioli J. (2015). Valved glaucoma drainage devices in pediatric glaucoma. *JAMA Ophthalmology*.

[4] Jacobson A., Besirli C. G., Bohnsack B. L. (2022). Outcomes of Baerveldt glaucoma drainage devices in pediatric eyes. *Journal of Glaucoma*.

[5] Grover D. S., Godfrey D. G., Smith O., Feuer W. J., Montes de Oca I., Fellman R. L. (2014). Gonioscopy-assisted transluminal trabeculotomy, ab interno trabeculotomy: technique report and preliminary results. *Ophthalmology*.

[6] Grover D. S., Smith O., Fellman R. L. (2015). Gonioscopy assisted transluminal trabeculotomy: an ab interno circumferential trabeculotomy for the treatment of primary congenital glaucoma and juvenile open angle glaucoma. *The British Journal of Ophthalmology*.

[7] Chen J., Wang Y. E., Quan A. (2020). Risk factors for complications and failure after gonioscopy-assisted transluminal trabeculotomy in a young cohort. *Ophthalmology Glaucoma*.

[8] Lehmann-Clarke L., Sadeghi Y., Guarnieri A., Sharkawi E. (2020). Gonioscopy-assisted transluminal trabeculotomy using an illuminated catheter for infantile primary congenital glaucoma. *American Journal of Ophthalmology Case Reports*.

[9] Song Y., Zhang X., Weinreb R. N. (2022). Gonioscopy-assisted transluminal trabeculotomy in primary congenital glaucoma. *American Journal of Ophthalmology Case Reports*.

[10] Aboalazayem F., Elhusseiny A. M., El Sayed Y. M. (2023). Gonioscopy-assisted transluminal trabeculotomy: a review. *Current Eye Research*.

[11] Shi Y., Wang H., Yin J. (2017). Outcomes of microcatheter-assisted trabeculotomy following failed angle surgeries in primary congenital glaucoma. *Eye*.

[12] Hu M., Wang H., Huang A. S. (2019). Microcatheter-assisted trabeculotomy for primary congenital glaucoma after failed glaucoma surgeries. *Journal of Glaucoma*.

[13] Papadopoulos M. E. B., Chiang M., Mandal A., Grajewski A. L., Khaw P. T. (2013). Section 5: Glaucoma surgery in children. *Childhood Glaucoma*.

[14] Elhusseiny A. M., El Sayed Y. M., El Sheikh R. H., Gawdat G. I., Elhilali H. M. (2019). Circumferential Schlemm’s canal surgery in adult and pediatric glaucoma. *Current Eye Research*.

