# Supplementary material for: Gonioscopy-Assisted Transluminal Trabeculotomy following Failed Glaucoma Surgery in Primary Congenital Glaucoma: One-Year Results
Source: Case Rep Ophthalmol Med. 2023 Jun 1;2023:6761408. doi: 10.1155/2023/6761408 (PMC10250098; doi:10.1155/2023/6761408)
Supplement: Supplementary Materials — Video: surgical video showing steps of gonioscopy-assisted transluminal trabeculotomy in a primary congenital glaucoma patient. [file 6761408.f1.docx]

Supplementary video

Download link
[https://wetransfer.com/downloads/cedf6bda1240986b04edf141dc97093220230515104708/ef4bdaaaa547e43862d4b1880626fcb020230515104727/c47b99](https://urldefense.com/v3/__https:/wetransfer.com/downloads/cedf6bda1240986b04edf141dc97093220230515104708/ef4bdaaaa547e43862d4b1880626fcb020230515104727/c47b99?trk=TRN_TDL_01&utm_campaign=TRN_TDL_01&utm_medium=email&utm_source=sendgrid__;!!N11eV2iwtfs!qoMU5N04wK4y6oLaEEf6I73FfGhGXNUxqNMWHGIzT578nfhHSkyGl6QWGh1m3_wlaMNVxP9YIv_gGm5ONyiRAfkTHtg%24)
